# Use of Fuzzy Analytic Hierarchy Process and Environmental Gini Coefficient for Allocation of Regional Flood Drainage Rights

**DOI:** 10.3390/ijerph17062063

**Published:** 2020-03-20

**Authors:** Dandan Zhang, Juqin Shen, Pengfei Liu, Qian Zhang, Fuhua Sun

**Affiliations:** 1 Business School, Hohai University, Nanjing 211100, China; qzhang@hhu.edu.cn; 2 College of Agricultural Engineering, Hohai University, Nanjing 210098, China; jqshen@hhu.edu.cn; 3 School of Instrument Science and Engineering, Southeast University, Nanjing 210096, China; 18252585090@163.com

**Keywords:** allocation of flood drainage rights, Delphi method, fuzzy analytic hierarchy process, environmental Gini coefficient

## Abstract

To solve the flood drainage conflict among different regions of the water basin when the flood occurs, it is of great significance to study the allocation of flood drainage rights. The allocation of flood drainage rights requires flood management departments to consider the influences of socioeconomic differences among different regions on flood control operations to realize sustainable development. Under the pattern of the total amount allocation of “watershed–administrative regions”, the evaluation index system of flood drainage rights allocation incorporated four aspects: natural conditions, level of social development, level of economic development, and technology and management. The fuzzy analytic hierarchy process (FAHP) was used to calculate the weight coefficient of each allocation index and the initial distribution’s proportion of the total amount in each region. Land area, population, gross domestic product (GDP), and sewage treatment capacity were selected as the evaluation indexes of the environmental Gini coefficient, and the environmental Gini coefficient method was used to evaluate and adjust the initial allocation of each region. Taking the allocation of flood drainage rights in the Taihu Basin as a case study, the final allocation results were obtained after initial allocation and feedback optimization. By evaluating the environmental Gini coefficient of each evaluation index, it is concluded that the final allocation could meet the requirements of fair allocation in each administrative region and be effectively implemented. Optimal allocation of the flood drainage rights in the Taihu Basin can contribute to overall flood control management, the reduction of flood disasters, and the stable development of society in the basin.

## 1. Introduction

Existing studies show that global warming would change the status quo of the global hydrological cycle by affecting rainfall, evaporation, runoff, soil moisture, etc., and causing the redistribution of water resources in time and space [1,2]. As temperatures rise, new precipitation patterns, rising sea levels, and changes in the frequency and intensity of storms are exacerbating flooding problems in many countries with relatively abundant water resources [3,4]. The Intergovernmental Panel on Climate Change (IPCC) has stated that global flooding would become more frequent and costs would increase [5,6].

For flood disasters, the minimization of losses in the whole basin is possible if the regions within the basin cooperate closely and realize joint regulation actively [7]. For example, when the highest flood level of the Thames meets the high water level, this confluence poses a threat to the lower reaches of the river, including the Greater London area. Therefore, flood control in the lower reaches has become one of the priorities for managing the Thames [8]. When the drainage demand of the upstream and downstream of the drainage basin exceeds the total allowable amount of flood drainage, if each region tries to discharge more floodwater to reduce the drainage pressure, and takes its adjacent region as the flood discharge gully, a series of disputes about cross-boundary flood drainage rights and the water environments would occur between the upstream and downstream regions. In Europe, flooding and industrial pollution in the lower Rhine are the main contributors to conflicts about water [9,10]. In 2011, climate change led to major flooding in Bangkok, Thailand. To prevent the flood from affecting the city center, the local government clashed with the public over the decision to open an upstream sluice [11,12,13]. The sustainable development of both the economy and society is likely to be impeded if the issue of flood discharges among different regions cannot be coordinated.

Over the years, China has attached great importance to the construction of water conservancy facilities and accumulated rich experience in flood control and drainage, but it still has some of the most severe flood disasters in the world [14,15,16]. In recent years, extreme weather events have been occurring frequently in many areas of China, leading to insufficient flood discharge capacities in the major rivers and to changes in runoff and confluence conditions. As a result, flood disaster and drainage problems in the upper and lower reaches of the river basin have become increasingly serious and more urgent requirements have been put forward for flood control and drainage in the basin [17,18]. When a flood disaster occurs, departments of water resource management comprehensively consider the drainage capacities of water conservancy projects, the distribution of rainfall, and the drainage demand in each region. From an economic perspective, these departments should consider the minimization of costs and expenses, as well as the maximization of profits. They should also pay attention to social, technical, demographic, and other factors involved in the total amount allocation process so as to achieve the appropriate fairness and efficiency [19,20].

Flood drainage rights refer to the right to discharge floodwater generated by a rainstorm. The distribution process of flood drainage rights is a multi-objective complex system related to the government, enterprises, people, and other aspects. The management and optimal allocation of flood drainage rights contribute to solving the drainage disputes between the upper and lower reaches of the river basin, reducing the flood damage to the whole basin, and ensuring the stable production of grain, sources of income to farmers, and regional development [21]. Further research is needed on the reasonable distribution of the total amount of flood drainage in a watershed–administrative region. From the perspective of systems science, this study combines subjective judgments with objective factors to establish a feedback optimal allocation model based on the fuzzy analytic hierarchy process (FAHP) and environmental Gini coefficient methods.

## 2. Literature Review

In addition to natural causes, vegetation destruction and river system changes caused by human activities are the main causes of flood disaster in many inland watersheds. Flood duration is an important factor that determines the formation and severity of a flood disaster [22]. Because of the locality of flood disasters, the unfairness caused by management measures should be reduced as much as possible in the flood management mode [23]. Schultz [24] conducted a study on how food production, water pollution, and shortages affected flood control measures and the formulations of schemes in emerging developing countries with different climatic and socioeconomic conditions. So far, research has focused on flood management and control [25,26,27], as well as early warning systems [28,29]. Studying urban drainage system construction, Verworn [30] discussed the responses of urban flood control mechanisms to rainstorms and floods. Sayers et al. [31] introduced several algorithms applied to multi-objective optimizations of flood risk intervention strategies for urban drainage networks and formulated improved methods to determine intervention strategies for reducing flood risks in urban environments. Vlotman et al. [32] offered insights into the integration of drainage, water quality, and flood management in rural, urban, and low-lying areas. Nagasaki University in Japan has successfully developed a flood control early warning and monitoring system that needs only data such as local rivers, water conservancy conditions, and rainfall that are input into a database and combined with topographical information. Such a system provides the necessary time for the proposal of disaster prevention plans. In addition to engineering measures, the United States, the United Kingdom, Japan, and other countries proposed the concept of non-engineering flood control measures in the middle of the 20th century to strengthen the flood control management intensities in flood areas by setting up detention basins [33,34]. Mascarenhas et al. [35] discussed detention basins constructed in a super-urban environment with rapid economic and population development. Elliott and Trowsdale [36] analyzed the effects of flood storage and diversions in detention basins, as well as their impacts on water quality and the surrounding environments. Xu [37] constructed spatial allocation and trading systems of the flood drainage rights in the Qinhuai River Basin as a new means of optimizing the compensation mechanisms of detention basins. Zhang et al. [20] established a bi-layer multi-objective optimization model for flood drainage rights from the perspective of fairness and efficiency, then configured the drainage rights in the six flood control areas of the Sunan Canal. The formulation of non-engineering countermeasures should take into account the economic benefits and stakeholder recommendations with a focus on sustainable development [38].

At present, the management of inter-regional flood drainage rights is implemented mainly from the perspective of hydrology. The social, economic, environmental, and resource differences among regions pose difficulties for the consideration of fairness and efficiency by the existing allocation models [39,40]. To realize sustainable development, the allocation of flood drainage rights requires flood management departments to consider the influences of social and economic differences between different regions, on the basis of which the joint operations of units in a river basin are carried out. Under the pattern of the total amount allocation of “watershed-administrative regions”, this study considered the social, economic, natural and technical attributes of the region, and constructed the evaluation index system of flood drainage rights allocation. Through the initial allocation and the feedback optimization, the allocation scheme can be effectively implemented.

## 3. Materials and Methods

### 3.1. Research Area

The Taihu Basin is located in the core region of the Yangtze River Delta at 30°5′ N and 119°8’ E. With a drainage area of 3.69 × 10^4^ km^2^, the basin is centered on Taihu Lake, with the Huangpu River as the main drainage channel is adjacent to the Yangtze River, East China Sea, Hangzhou Bay, and Qiantang River, which are to the north, east, and south, respectively, and bounded in the west by the Maoshan and Tianmu Mountains. The Taihu Basin is a subtropical monsoon climate zone in China and has a topography characterized by high surrounding and low central areas. The terrain of the basin is flat, the plain area accounts for about 80% of the total basin area, and the river system is well developed. The total length of the regional channel is about 12 × 10^4^ km and the density is about 3.25 km/km^2^, which is typical of a plain river network area in China [41].

**Figure 1:**
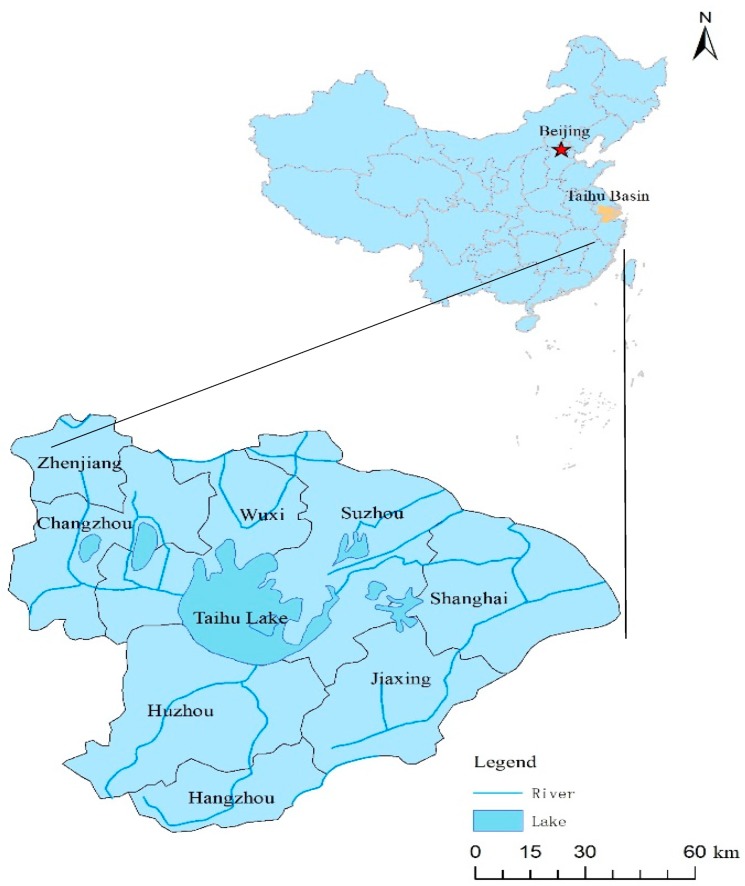
Administrative regions in Taihu Basin.

The administrative divisions belong to the provinces of Jiangsu, Zhejiang, and Anhui, as well as to Shanghai City. There are eight cities in the basin: Wuxi, Changzhou, Suzhou, Zhenjiang, Hangzhou, Jiaxing, Huzhou, and Shanghai (Figure 1). The Taihu Basin is one of the regions with the fastest economic development and the highest levels of urbanization in China. In 2017, for example, the land area of the basin accounted for 0.4% of the national land area. The basin’s population and gross domestic product (GDP) accounted for 4.4% and 9.8% of the country’s population and GDP, respectively.

With significant inter-annual variation and uneven annual rainfall distribution, the average annual precipitation in the basin is 1177 mm. June and July are known as the “plum rain” period, which has a long duration and a large amount of rainfall, which can easily cause basin floods. August to October is a period of frequently occurring typhoons. The rainstorms due to typhoons are concentrated and strong, which can easily cause regional floods [42]. With the rapid economic and social development in China, the Taihu Basin began to suffer from frequent floods in the 1980s. The changes in the river network structures and water levels, as well as the increase in precipitation, caused by human activities are the main reasons for such frequency [43,44].

### 3.2. Construction of Index System

According to the basin’s natural characteristics and type of administrative, the pattern of the total amount allocation of flood drainage rights is determined as a watershed–administrative region. Having its own natural, social, economic, and technological attributes, each control unit needs complete information about its region.

When the total amount allocation index is chosen, the actual situation of each allocation unit should be considered comprehensively for the realization of the principle of efficiency based on fairness. The specific principles for the selection of the allocation indexes are as follows: (1) The principle of comprehensiveness. All evaluation indicators should comprehensively reflect the current status of each control unit in social, economic, environmental, and water resources technical management. Through a systematic description of all aspects, the index system should be hierarchical and unrepeatable, i.e., one part of the evaluation index should have a subordinate relationship with another part. (2) The principle of representativeness. For the FAHP, excessive or no representativeness of the allocation indicators would result in unfair and inefficient allocation. Therefore, the selected indicators should be concise and representative when representing the actual situation of the control unit. (3) The principle of comparability. The establishment of the index system should consider horizontal comparisons between regions, so the comparability of the status quo between regions should be considered. (4) The principle of operability. The selected allocation index data are easy to obtain and can be quantified to facilitate the generation of a judgment matrix.

According to the principle of index selection, the expert consultation method is adopted to screen the indexes and determine the four factors, which are natural conditions, level of social development, level of economic development, and technology and management. These factors are directly related to the total amount allocation of each control unit [45]. The above four factors constitute the criterion layer of the FAHP index system. Each factor contains several specific indicators, which constitute the index layer of the FAHP. The selection of the index in the index layer must still follow the principles of comprehensiveness and operability. The specific indicators are selected as shown in Table 1. The water production coefficient refers to the total amount of water resources to annual rainfall. The spatial distribution difference index refers to the ratio of the disposable incomes of urban residents to the net incomes of rural residents.

### 3.3. Normalization of Data

To eliminate the differences in the measurement units and order of magnitudes among evaluation indexes, this study normalized the indexes. For each allocation index, the contribution rate was analyzed according to the statistical data of each administrative region and the index value of each administrative region was normalized to the range (0,1). Consequently, the sum of the normalization results of each administrative region equals 1.

According to the type of correlation between the factors in the criterion layer (B) and the index layer (C), the indicators were divided into positive and negative. Different normalization methods were adopted for these two types of indicators, as follows:

(1) $$\{\begin{array}{l} \begin{array}{cc} \begin{array}{cc} positive & indicators: \end{array} & \begin{array}{cc} z_{uq}=\frac{o_{uq}}{\sum_{q=1}^{m}o_{uq}} & \end{array} \end{array}\\ \begin{array}{cc} negative & indicators: \end{array}e_{uq}=1-\frac{o_{uq}}{\sum_{q=1}^{m}o_{uq}},z_{uq}=\frac{e_{uq}}{\sum_{q=1}^{m}e_{uq}} \end{array}u=1,2,\cdots n;q=1,2,\cdots m$$

where $z_{uq}$ is the normalized value of allocation index u in administrative region q, $o_{uq}$ is the original value of index u in region q, $e_{uq}$ is the influence value of index u in region q, m is the number of administrative regions, and n is the number of allocation indexes.

### 3.4. Allocation Method of Flood Drainage Rights

For the total amount allocation of flood drainage rights in a watershed–administrative region, the FAHP method was used to calculate the weight coefficient of each allocation index and the initial allocation of the total amount, then the environmental Gini coefficient method was used to evaluate and adjust the initial allocation of each control unit.

#### 3.4.1. Initial Allocation Based on FAHP

##### Generation of Judgment Matrix

The FAHP is a method that combines fuzzy mathematical theory with the analytic hierarchy process (AHP). To obtain the weight of each index more accurately, fuzzy mathematics was introduced to construct the fuzzy consistent judgment matrix through the comparison of two elements, thereby avoiding the difficulty in adjusting the consistency of the comparison judgment matrix in AHP [46,47,48].

In this study, a questionnaire was designed according to the total amount allocation index system, then seven experts were invited to form a panel. Following the Delphi method, each expert judged the degrees of the importance of factors B_1_, B_2_, B_3_, and B_4_ in the criterion layer to the target layer. The importance degrees were assigned values of 1–9, then each element was compared to each of the other elements and scored. The values of the importance scale of the judgment matrix are shown in Table 2.

When the degrees of the importance of the four factors in the criterion layer to the target layer (A) were compared, the degrees of the influence of factors $B_{u}$ and $B_{v}$ on the target layer were also compared. $K_{uv}$ is the ratio of the relative importance of $B_{u}$ to $B_{v}$ and is calculated by $K_{uv}=\frac{1}{K_{vu}}$. Finally, the judgment matrix of the criterion layer to the target layer was obtained:

(2) $$K\left(A-B\right)=\left[\begin{array}{llll} k_{11} & k_{12} & k_{13} & k_{14}\\ k_{21} & k_{22} & k_{23} & k_{24}\\ k_{31} & k_{32} & k_{33} & k_{34}\\ k_{41} & k_{42} & k_{43} & k_{44} \end{array}\right]$$

Each expert similarly judged the degrees of the influences of indicators C_1_, C_2_, C_3_, and C_4_ on factor B_1_; C_5_, C_6_, C_7_, C_8_ on B_2_; C_9_, C_10_, C_11_, C_12_, C_13_ on B_3_; C_14_, C_15_, C_16_, C_17_, and C_18_ on B_4_. The judgment matrixes $K\left(B_{1}-C\right)$, $K\left(B_{2}-C\right)$, $K\left(B_{3}-C\right)$ and $K\left(B_{4}-C\right)$ were generated from the results of the judgment.

##### Establishing the Fuzzy Positive Reciprocal Matrix

Fuzzy numbers are fuzzy sets of normalized and convex sets. The membership functions of fuzzy numbers must meet the following conditions: (1) normalized fuzzy subset; (2) convex fuzzy subset; (3) continuous section [49]. Triangular fuzzy numbers can be expressed as F=(i,j,k) with i≤j≤k. When i>0, F is a positive triangular fuzzy number. The membership function $\delta_{\tilde{F}}\left(x\right)$ of a triangular fuzzy number F is defined as:

(3) $$\delta_{\tilde{F}}\left(x\right)=\{\begin{array}{c} \frac{x-i}{j-i},i\leq x\leq j\\ \frac{k-x}{k-j},j\leq x\leq k\\ 0,other \end{array}$$

From the expert scoring’s results, the judgment matrix of each level of indicators was obtained and the judgment matrix was transformed into a fuzzy positive reciprocal matrix according to the relative importance assessment criteria (Table 3).

The fuzzy positive reciprocal matrix is expressed as follows:

(4) $$F^{t}={\left\lfloor F_{uv}\right\rfloor }_{n\times n}u,v=1,2,\cdots n$$

where $F^{t}$ is the fuzzy positive reciprocal matrix of the t-th expert and $F_{uv}$ is the comparison value of the importance of the t-th expert for the index u relative to the index *v*.

If u=v, then $F_{uv}=\left(1,1,1\right)$. If u≠v and assuming that u is absolutely important than v, then $F_{uv}=\left(7,9,9\right)$ and $F_{uv}=\frac{1}{F_{vu}}=\left(\frac{1}{7},\frac{1}{9},\frac{1}{9}\right)$.

##### Calculation of Fuzzy Weight

**Figure 2:**
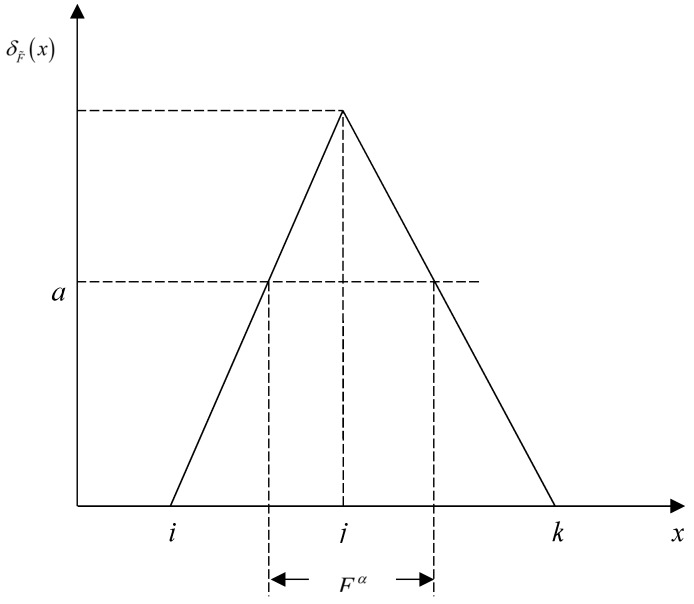
α—cut set of triangular fuzzy number *F^α^*.

An α-cut set is a method of turning a fuzzy set into a scoped set, as shown in Figure 2. The definition of an α-cut set of a triangular fuzzy number F is:

(5) $$F^{\alpha }=\left[\left(j-i\right)\alpha +i,j,k-\left(k-j\right)\alpha \right],0\leq \alpha \leq 1$$

where $F^{\alpha }$ is the set of all elements that belong to F to a degree greater than or equal to α.

(1) Set α=1, then according to the α-cut set, calculate the positive reciprocal matrix $F_{r}^{t}={\left\lfloor F_{uvr}\right\rfloor }_{n\times n}$ of the t-th expert, calculate the eigenvector of $F_{m}^{t}$, and normalize the eigenvector to obtain the weight $W_{m}^{t}$ of $F_{m}^{t}$.

(2) Set α=0, then calculate the upper and lower limit positive reciprocal matrixes, which are $F_{r}^{t}={\left\lfloor F_{uvr}\right\rfloor }_{n\times n}$ and $F_{l}^{t}={\left\lfloor F_{uvl}\right\rfloor }_{n\times n}$, respectively, of the t-th expert, and calculate their respective weights, $W_{r}^{t}$ and $W_{l}^{t}$.

(3) According to $W_{l}^{t}$, $W_{m}^{t}$, and $W_{r}^{t}$, the fuzzy weight value $W_{u}^{t}=\left(W_{l}^{t},W_{m}^{t},W_{r}^{t}\right)$ of the t-th expert to the u-th index can be obtained.

(4) The average method is used to integrate the fuzzy weight values of all experts:

(6) $$\bar{W_{u}}=\frac{1}{t}\left(W_{u}^{1}⊕W_{u}^{2}⊕\wedge ⊕W_{u}^{t}\right)$$

where $\bar{W_{u}}$ is the fuzzy weight value of the u-th index, $W_{u}^{t}$ is the fuzzy weight value of the t-th expert on the u-th index, and t is the number of experts.

According to the properties and expansion principle of fuzzy numbers, if $F_{1}=\left(i_{1},j_{1},k_{1}\right)$ and $F_{2}=\left(i_{2},j_{2},k_{2}\right)$, then the algebraic algorithm of the triangular fuzzy numbers is $F_{1}⊕F_{2}=\left(i_{1}+i_{2},j_{1}+j_{2},k_{1}+k_{2}\right)$ and $F_{1}⊗F_{2}=\left(i_{1}\times i_{2},j_{1}\times j_{2},k_{1}\times k_{2}\right)$.

By the simple barycenter method, the fuzzy weight of index u is converted into $\delta_{u}$ by:

(7) $$\delta_{u}=\frac{i_{u}+j_{u}+k_{u}}{3}$$

##### Calculation of Initial Allocation Weight

The statistical data of each administrative region were collected and normalized. The normalized result was $z_{uq}$. The initial allocation weight $\eta_{q}$ of the administrative region q is calculated by:

(8) $$\eta_{q}=\sum_{u=1}^{n}w_{u}z_{uq}u=1,2,\cdots ,n$$

where $\eta_{q}$ is the initial allocation weight coefficient of the q-th administrative region, $w_{u}$ is the weight of the u-th allocation index, and $z_{uq}$ is the normalization result of the u-th index in the q-th region.

#### 3.4.2. Adjustment of Initial Allocation According to Environmental Gini Coefficient

##### Environmental Gini Coefficient Model

The selection of the allocation index of the environmental Gini coefficient should be combined with a particular region’s levels of social and economic development [50]. Referring to Xu [51] and Xue et al. [52], this study selected land area (L), population (P), GDP (G), and sewage treatment capacity (S) as the evaluation indexes of the environmental Gini coefficient, which was calculated by combining the indexes with the weight of the initial drainage rights allocation. Taking L as an example, the specific calculation procedure is:

(1) The allocation weight $\gamma_{q}$ per unit land area of region q is calculated by Equation (9) and the administrative regions are ranked from smallest to largest $\gamma_{q}$:

(9) $$\gamma_{q}=\frac{\eta_{q}}{L_{q}}$$

where $\gamma_{q}$ is the weight of flood drainage rights allocated to the unit land area of region q and $L_{q}$ is the land area of region q.

(2) The cumulative sum of $\gamma_{q}$ is taken as the cumulative percentage of allowable displacement $Y_{q}$. The cumulative percentage of land area $X_{q}$ is calculated according to the order in Step (1) above. With X and Y as the horizontal and vertical axes, respectively, the Lorenz curve is drawn and the Gini coefficient of the land area is calculated by:

(10) $$Y_{q}=Y_{\left(q-1\right)}+\gamma_{q}$$

(11) $$X_{q}=X_{\left(q-1\right)}+L_{q}/\sum_{q=1}^{m}L_{q}$$

(12) $$G=1-\sum_{q=1}^{m}\left(X_{q}-X_{\left(q-1\right)}\right)\left(Y_{q}-Y_{\left(q-1\right)}\right)$$

where G is the environmental Gini coefficient corresponding to the evaluation index, $X_{0}=0$, and $Y_{0}=0$. The environmental Gini coefficients of population (P), GDP (G), and sewage treatment capacity (S) are similarly obtained.

##### Adjustment Process of Initial Allocation

(1) Judgment of fairness of initial allocation

For the Gini coefficient, a value of 0.4 is used internationally as an alert value to judge the fairness of distribution. If the Gini coefficient is less than or equal to 0.4, then the distribution is fair; otherwise, it is unequal [53,54,55]. The alert value can be adjusted for different requirements of the total amount allocation. If $\max\left\{G_{\varphi }\right\}\leq 0.4$, then the initial allocation weight of the total amount does not need to be adjusted.

Since the initial allocation model of the FAHP cannot meet the fairness of each administrative region at any one time, the allocation scheme cannot be implemented effectively. Therefore, this study determined the treatment of the three situations under the total amount allocation thus:

① If there were two or more cases in which $G_{\varphi }\geq 0.4\left(\varphi =L,P,G,S\right)$, then the subjectivity of the initial allocation results was considered to be too great and returning to the FAHP model to re-judge the fuzzy weight would be necessary.

② If there was only one case in which $G_{\varphi }\geq 0.4$, then the environmental Gini coefficient could be reduced by adjustments made according to the constraints.

③ If there was any case in which $G_{\varphi }<0.4$, then the allocation result was considered to be fair and the total allocation scheme of the flood drainage rights in the basin was determined under the condition that most control units accepted the allocation scheme.

**Figure 3:**
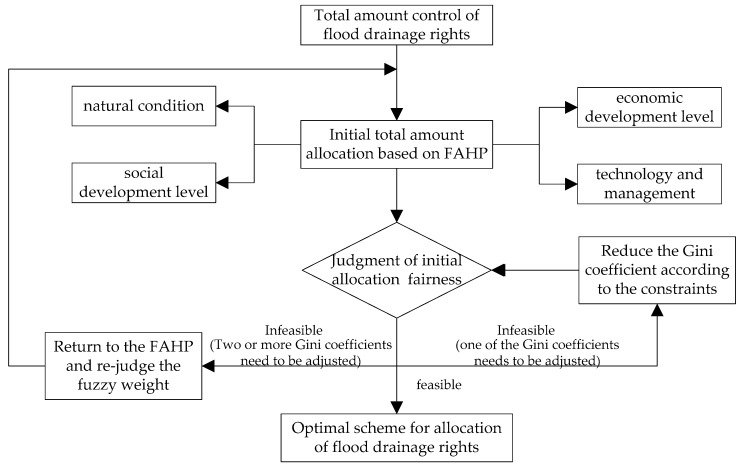
Technical route of feedback optimization.

The technical route of the feedback optimization of the initial allocation model was determined as shown in Figure 3.

(2) Constraints on adjustment of initial allocation

When $\max\left\{G_{\varphi }\right\}>0.4$, then the initial allocation weight was adjusted by:

(13) $$\{\begin{array}{c} 0<G_{\varphi }^{a}\leq 0.4\\ \begin{array}{l} \max\left\{G_{\varphi }^{a}\right\}\to 0.4\\ \sum G_{\varphi }^{a}\leq \sum G_{\varphi }^{0} \end{array}\\ \eta_{q}^{a}>0\\ \sum_{q=1}^{m}\eta_{q}^{a}=1 \end{array},\varphi =L,P,G,S$$

where $G_{\varphi }^{a}$ is the Gini coefficient of the φ-th evaluation index after the a-th adjustment, $G_{\varphi }^{0}$ is the Gini coefficient of the φ-th evaluation index of the initial allocation, and $\eta_{q}^{a}$ is the weight of the flood drainage rights of the q-th region after the a-th adjustment.

## 4. Results and Discussions

### 4.1. Index Normalization Results

**Figure 4:**
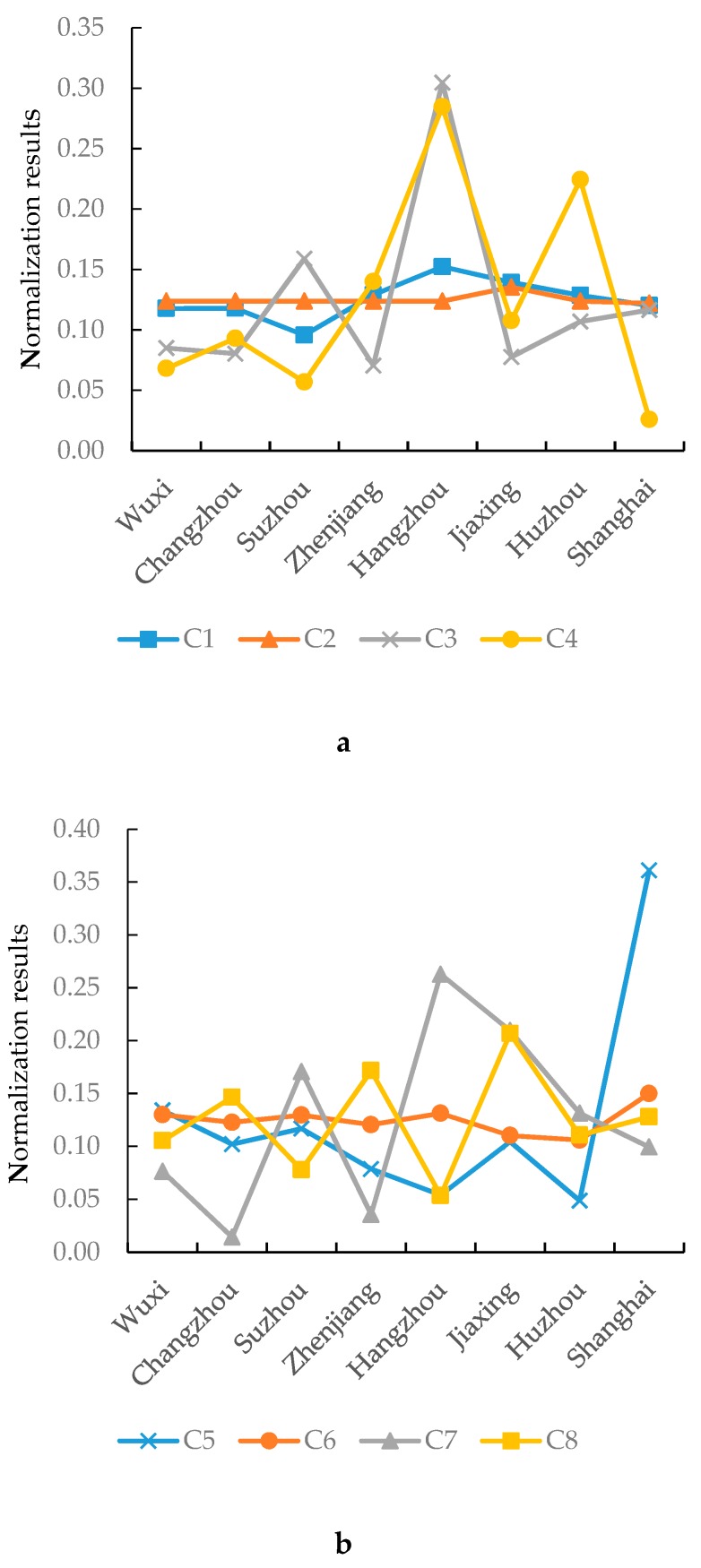

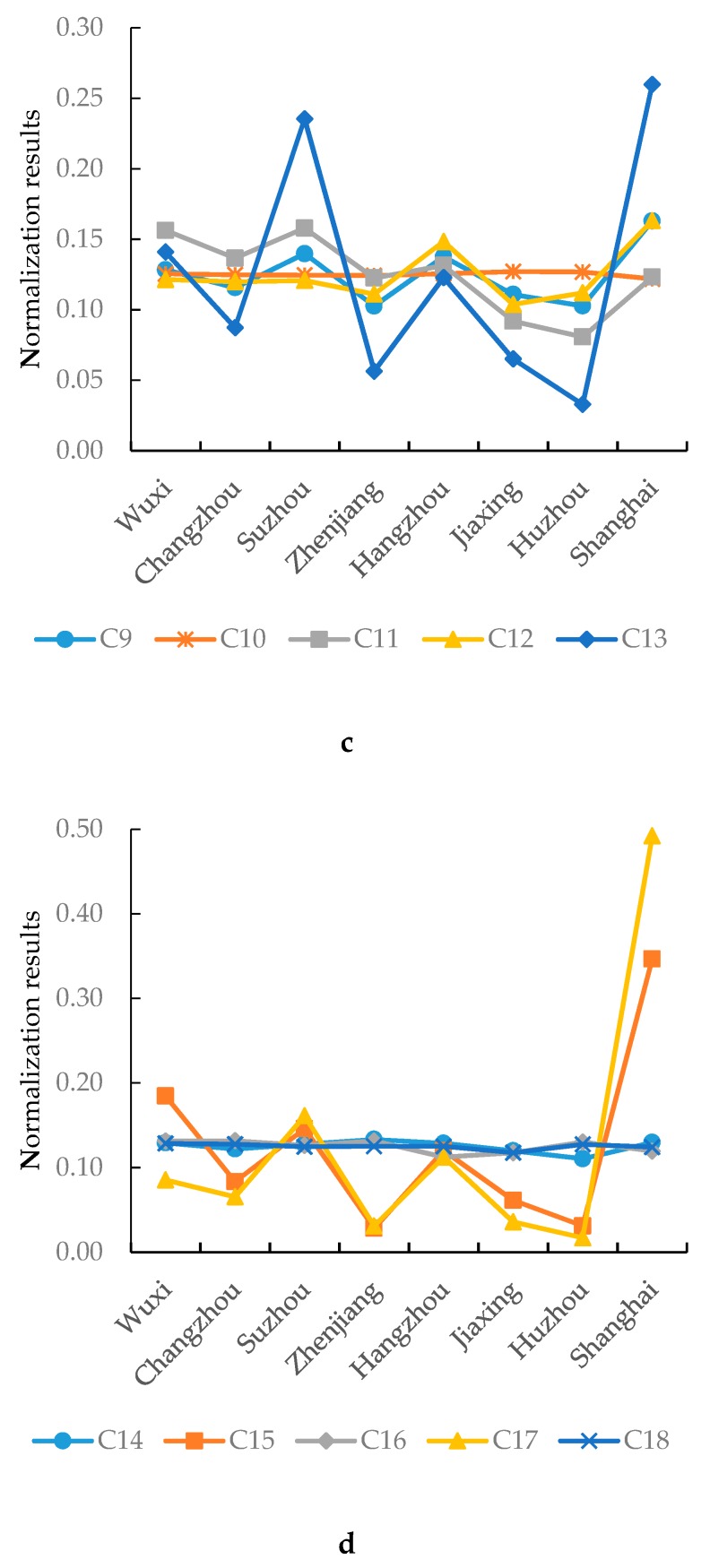
Normalization results of indexes in each region; (**a**) Natural condition; (**b**) Social development level; (**c**) Economic development level; (**d**) Technology and management.

The original values of each index were obtained from the 2017 statistical yearbook, water conservancy yearbook, and water resources bulletin of each region. The indexes were normalized by Equation (1) to obtain the normalization results of each index in each factor (Figure 4).

In terms of natural conditions, the distribution of water production coefficient (C_1_) and drainage density (C_2_) were relatively average. Hangzhou had more land area (C_3_), whereas Zhenjiang had less. Also, Hangzhou was relatively richer in water resources per capita (C_4_), whereas Shanghai was relatively poorer.

From the perspective of the level of social development, the population density (C_5_) of Shanghai was the highest because of its large migrant population, whereas that of Huzhou was the lowest. The urbanization rates (C_6_) of all regions were relatively close. Hangzhou had the highest natural growth rate of population (C_7_), whereas Changzhou had the lowest. The cultivated land density (C_8_) of Jiaxing was the highest, whereas that of Hangzhou was the lowest.

From the perspective of the level of economic development, the distribution of disposable income per capita (C_9_), GDP per capita (C_11_), and the proportion of the output value of tertiary industry (C_12_) were similar. The spatial distribution difference index (C_10_) among different regions was relatively average. Shanghai had the highest industrial added value (C_13_), followed by Suzhou, whereas Huzhou had the lowest.

From the perspective of technology and management, the distribution of the water consumption per unit of GDP (C_14_), green coverage rate of the built-up areas (C_16_), and sewage treatment rate (C_18_) were close and evenly distributed. The distribution of the drainage pipe length in the built-up areas (C_15_), and sewage treatment capacity (C_17_) were close. Shanghai had the highest values of both.

### 4.2. Initial Allocation of Flood Drainage Rights

#### 4.2.1. Determination of Index Weight

From the questionnaires given to the seven experts, the judgment matrix of each expert was obtained (Table 4). Because of space constraints, only a portion of the questionnaire’s results from the first expert has been listed. The information of the seven experts is shown in Table 5.

As per Table 3, the opinions of the first expert were converted into a fuzzy positive reciprocal matrix:

$$F^{1}=\left[\begin{array}{cccc} \left(1,1,1\right) & \left(\frac{1}{3},\frac{1}{5},\frac{1}{7}\right) & \left(1,\frac{1}{3},\frac{1}{5}\right) & \left(\frac{1}{5},\frac{1}{7},\frac{1}{9}\right)\\ \left(3,5,7\right) & \left(1,1,1\right) & \left(1,3,5\right) & \left(\frac{1}{3},\frac{1}{5},\frac{1}{7}\right)\\ \left(1,3,5\right) & \left(1,\frac{1}{3},\frac{1}{5}\right) & \left(1,1,1\right) & \left(1,\frac{1}{3},\frac{1}{5}\right)\\ \left(5,7,9\right) & \left(3,5,7\right) & \left(1,3,5\right) & \left(1,1,1\right) \end{array}\right]$$

The weights of $F_{m}^{1}$, $F_{l}^{1}$, and $F_{r}^{1}$ were calculated according to the a-cut set, i.e., the fuzzy weight values of the factors in the criterion layer:

$$W_{1}^{1}=\left[\begin{array}{ccc} \begin{array}{l} 0.211\\ 0.393\\ 0.417\\ 0.792 \end{array} & \begin{array}{l} 0.082\\ 0.363\\ 0.202\\ 0.906 \end{array} & \begin{array}{l} 0.049\\ 0.322\\ 0.137\\ 0.935 \end{array} \end{array}\right]$$

The fuzzy weights ($W_{1}^{2}$, $W_{1}^{3}$, …, $W_{1}^{6}$) of the other six experts’ opinions on the factors of the criterion layer were similarly calculated. By Equation (6), the fuzzy weight values of all experts were integrated and the fuzzy weight matrix of the criterion layer was obtained:

$$\bar{W}=\left[\begin{array}{ccc} \begin{array}{l} 0.252\\ 0.295\\ 0.244\\ 0.208 \end{array} & \begin{array}{l} 0.212\\ 0.379\\ 0.166\\ 0.242 \end{array} & \begin{array}{l} 0.195\\ 0.419\\ 0.134\\ 0.252 \end{array} \end{array}\right]$$

By Equation (7), the fuzzy weight value of the criterion layer was converted into a single value. As with the algorithm of the criterion layer, the single weight value of the index layer was obtained. The single weight values of the judgment matrixes $K\left(B_{1}-C\right)$, $K\left(B_{2}-C\right)$, $K\left(B_{3}-C\right)$, and $K\left(B_{4}-C\right)$ were multiplied by the single weight values of the corresponding factors in K(A−B) to obtain the weight value of the index layer (C) to the target layer (A). The final results are shown in Table 6.

#### 4.2.2. Results of Initial Allocation of Administrative Regions

In accordance with the single weight values of K(A−C) obtained from Table 6 and the normalization treatment results of the indexes in each administrative region, the initial allocation results of the flood drainage rights of each administrative region can be calculated by the FAHP method, as shown in Table 7. The initial allocation weights of the allocated drainage rights in Wuxi, Changzhou, Suzhou, Zhenjiang, Hangzhou, Jiaxing, Huzhou, and Shanghai are 0.121, 0.107, 0.127, 0.104, 0.137, 0.119, 0.100, and 0.184, respectively.

### 4.3. Adjustment of Initial Allocation of Flood Drainage Rights

#### 4.3.1. Environmental Gini Coefficient of Initial Allocation

With the index of land area taken as an example and the basic data, the flood drainage rights per unit land area in eight regions were calculated and sorted (Table 8). The ranking of the results shows that the flood drainage rights per unit of land area from the lowest to the highest are Hangzhou, Suzhou, Huzhou, Shanghai, Wuxi, Changzhou, Zhenjiang, and Jiaxing.

**Figure 5:**
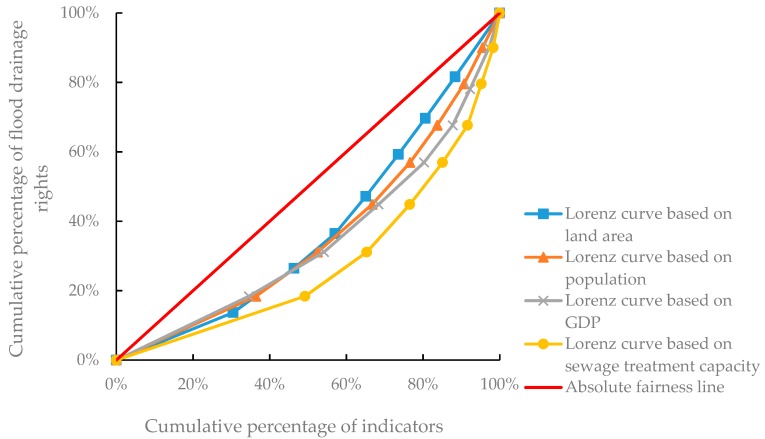
Lorenz curve of each evaluation index.

The Gini coefficient $G_{L}$ of the initial allocation of the flood drainage rights based on land area was calculated to be 0.250 while the environmental Gini coefficient $G_{P}$ based on population was 0.286, the environmental Gini coefficient $G_{G}$ based on GDP was 0.315, and the environmental Gini coefficient $G_{S}$ based on sewage treatment capacity was 0.439. $G_{L}$, $G_{P}$, and $G_{G}$ were all less than 0.4 within the range of equitable allocation, whereas $G_{S}$ was greater than 0.4, which is beyond the range of distributive equity. The corresponding Lorenz curve is shown in Figure 5. The curve of sewage treatment capacity has a larger arc, the area enclosed by the absolute fairness line is large, and the allocation fairness is poor.

#### 4.3.2. Final Allocations of the Regions

According to the Gini coefficients of the initial allocation, the environmental Gini coefficient based on sewage treatment capacity is greater than 0.4. The initial allocation scheme was optimized and adjusted according to the environmental Gini coefficients of the initial allocation and the adjustment method. Without any changes made to the weight ordering, the final allocation results of the total amount allocation were obtained, as shown in Table 9.

**Figure 6:**
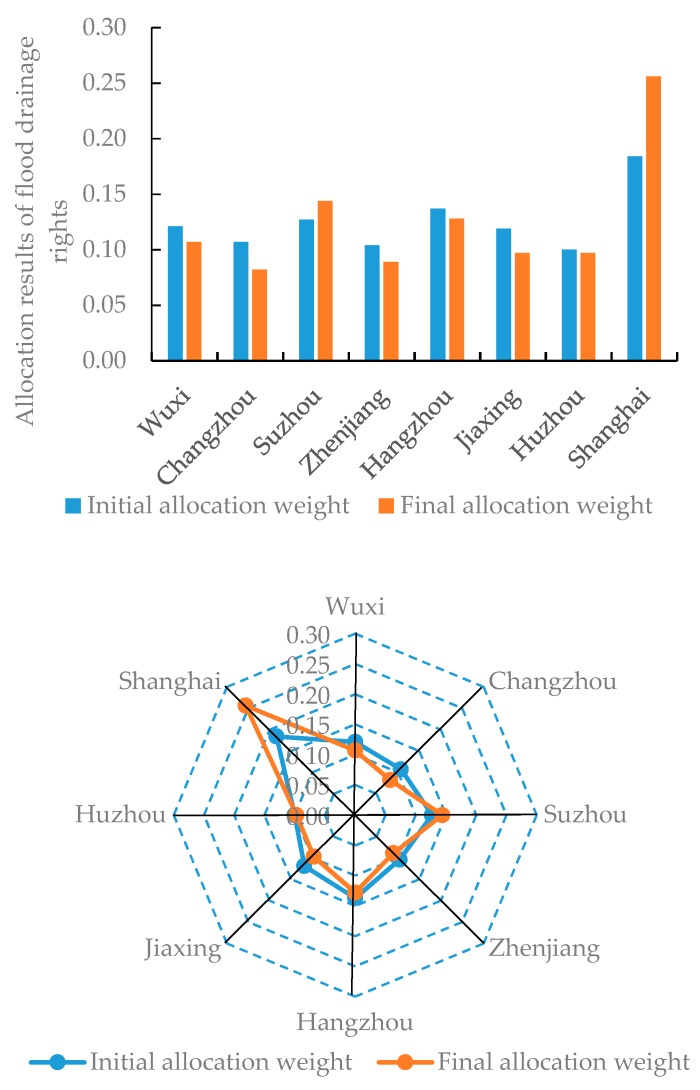
Allocation of flood drainage rights in each administrative region.

According to the inter-regional allocation results of the flood drainage rights in the Taihu Basin, the top three administrative regions are Shanghai, Suzhou, and Hangzhou. The final allocation proportions of the flood drainage rights are 0.256, 0.144, and 0.128, which account for 52.8% of the whole basin. The allocation of flood drainage rights in each administrative region is shown in Figure 6.

#### 4.3.3. Environmental Gini Coefficient of Final Allocation

**Figure 7:**
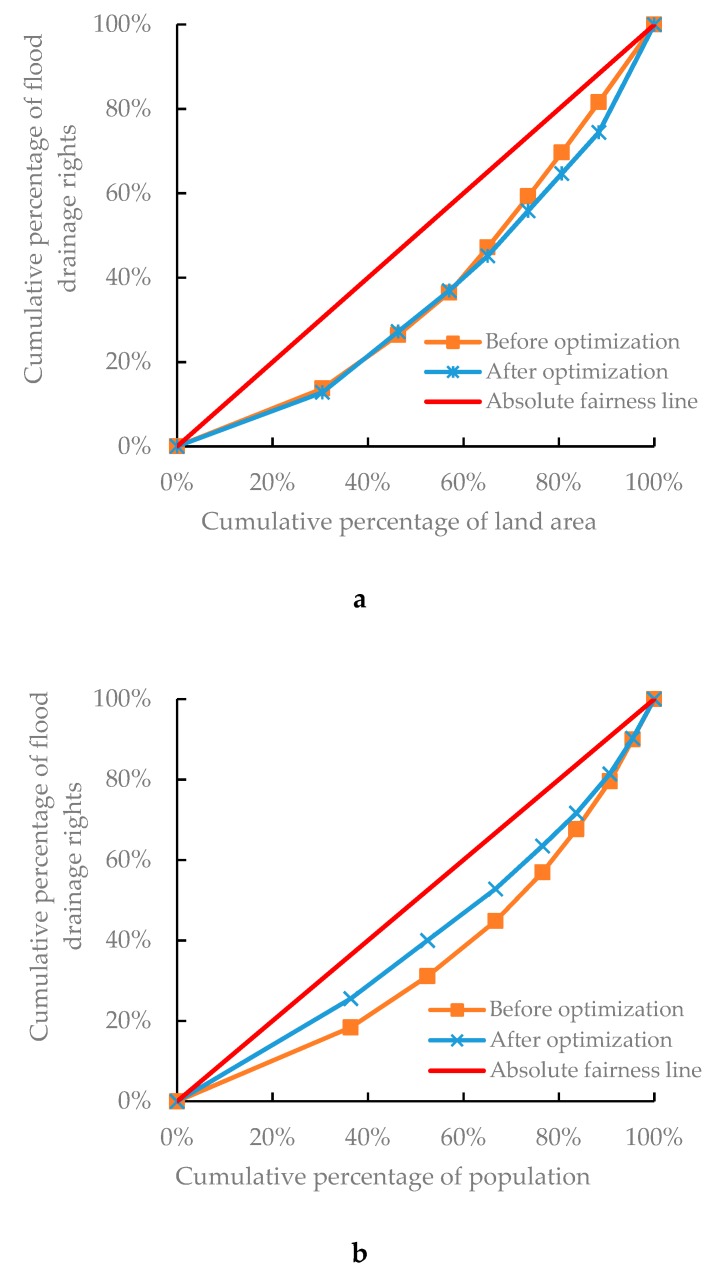

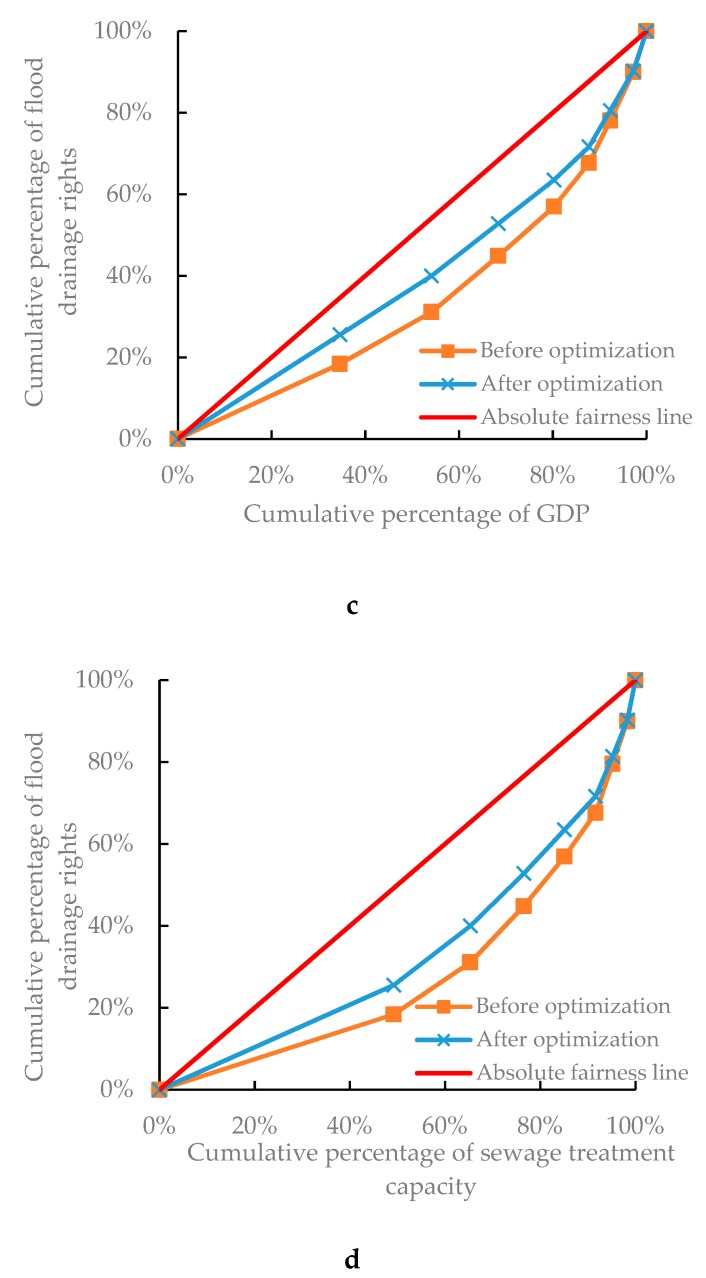
Lorenz curves of each index before and after optimization; (**a**) Lorenz curve based on land area; (**b**) Lorenz curve based on total population; (**c**) Lorenz curve based on GDP; (**d**) Lorenz curve based on sewage treatment capacity.

The environmental Gini coefficient of each evaluation index was obtained after feedback optimization by the environmental Gini coefficient method. Table 10 shows the environmental Gini coefficient of each index before and after optimization and Figure 7 shows the Lorenz curve of each index. The optimized results show that:

(1) After optimization, the environment Gini coefficients $G_{P}$, $G_{G}$, and $G_{S}$ all decrease, the sum of the Gini coefficient also decreases, so the degree of fairness increases.

(2) The environmental Gini coefficient $G_{L}$ based on land area becomes larger and weakens, to some extent, the influence of land area on the allocation of flood drainage rights. This weaker influence contributes to the implementation of the allocation scheme.

(3) After optimization, the environmental Gini coefficient $G_{S}$ based on sewage treatment capacity becomes 0.336, which is lower than 0.4, with a high level of fairness. The optimization results contribute to promoting the improvement of water resources technology and management.

### 4.4. Analysis of Allocation Results

Considering the natural environment, economic development and other factors, the rapid development of social economy leads to a high demand for the allocation of flood drainage rights. For the area with high economic development level, the higher recovery cost of flood disaster loss will lead to the incline of drainage right distribution to the high-speed development area, which is consistent with the view of Shen et al. [56].

The results of the allocation show that the order of the allocation weights of the flood drainage rights from highest to lowest is Shanghai, Suzhou, Hangzhou, Wuxi, Jiaxing, Huzhou, Zhenjiang, and Changzhou. The main reasons for the higher allocations of flood drainage rights in some regions are the following: (1) The economy is already relatively developed and continues to develop rapidly. The tertiary industry occupies a relatively large proportion of the GDP. Within the Taihu Basin, Shanghai ranks first in GDP while Suzhou and Hangzhou rank second and third, respectively. In addition, the output value of the tertiary industry in Shanghai ranks the first, the proportion of the output value of the tertiary industry in the GDP is as high as 69.18%, and the proportion of the flood drainage rights allocated is 25.6%. Therefore, to ensure the efficiency of allocation, the higher the level of economic development, the more flood drainage rights are allocated. (2) The population density is large and the degree of urbanization is high. For example, the population densities of Shanghai, Suzhou, and Wuxi are relatively large, with 3813 people/km^2^, 1234 people/km^2^, and 1416 people/km^2^, respectively. Their urbanization rates are more than 75%. Compared to areas with smaller population densities and lower urbanization levels, areas with faster urbanization rates require the allocation of more flood drainage rights. (3) The land areas of the cities are large. Generally speaking, the larger the land area, the greater is the demand for the allocation of flood drainage rights. Shanghai, Suzhou, and Hangzhou have larger land areas, so the allocations of flood drainage rights are higher. (4) The levels of technology and management are relatively high. In Shanghai, Suzhou, Hangzhou, and Wuxi, the water consumption per unit of GDP is less than 30 m^3^ while the built-up areas have dense drainage network distributions and strong sewage treatment capacities, which reflect the high levels of public service guarantees for urban construction and the high levels of technical support for drainage management. In this case, more flood drainage rights are allocated.

Jiaxing, Huzhou, Zhenjiang, and Changzhou are regions where the allocations of flood drainage rights are less than 10% because the regional economic aggregates are relatively small, the total populations are small, and the proportions of the urban populations are low. Moreover, the levels of technology and management in these regions are limited, so the proportions of flood drainage rights allocated to these areas are lower.

## 5. Conclusions

This paper proposes the pattern of watershed–administrative region distribution based on the principle of fairness. By using the fuzzy analytic hierarchy process (FAHP), the natural conditions, level of social development, level of economic development, and technology and management in each administrative region within the basin were taken as the four factors relevant to the design of an evaluation index system for the allocation of flood drainage rights and the initial allocation model for the total amount of flood drainage rights. The environmental Gini coefficient was taken as the basis for judging the fairness of the initial allocation. To rectify any unfair allocation results, the optimization technology route of the initial allocation results was determined and the feedback optimization model was established. For the allocation model, the Taihu Basin was used as the object of this study and the initial allocation of the flood drainage rights of each administrative region was carried out. After feedback optimization, the top four administrative regions for the flood allocation weights of the drainage rights were Shanghai, Suzhou, Hangzhou, and Wuxi. Jiaxing, Huzhou, Zhenjiang, and Changzhou had relatively small proportions of allocation.

The contributions of this paper are: (1) the selected indicators, which are closely related to the allocation of flood drainage rights and can be quantified, reflect the social, economic, and natural attributes of the regions. The data can be easily obtained from statistical yearbooks; (2) by the use of the FAHP and the environmental Gini coefficient method, the total amount allocation model of flood drainage rights has been optimized. The obtained allocation scheme is reasonable and can incorporate the differences in the social, economic, environmental, and technological management of each administrative region while ensuring its healthy development. The scheme has a certain reference value for the total allocation of flood drainage rights in other basins and regions.

The dynamic relationship between humans, nature, and the environment is influenced by social, political, and economic systems, which in turn are mediated and regulated through “governance” processes [57]. With the increasing attention being given to the issue of flood drainage rights, the management of inter-regional flood drainage rights will become more comprehensive and systematic in the future. The evaluation index system for the allocation of flood drainage rights should be further expanded and the relevant data should be improved based on the actual situation. On the basis of the hydrological conditions, social and economic statuses, and development requirements in the basin, a real-time allocation and management platform for flood drainage rights can be constructed. All this cannot be separated from the cooperation between the basin and the region.

## Figures and Tables

**Table 1:** Evaluation index system of flood drainage rights allocation.

Target Layer	Criterion Layer	Index Layer
Distributable flood drainage rights (A)	Natural conditions (B_1_)	Water production coefficient (C_1_)
		Drainage density (C_2_)
		Land area (C_3_)
		Water resources per capita (C_4_)
	Social development level (B_2_)	Population density (C_5_)
		Urbanization rate (C_6_)
		Natural growth rate of population (C_7_)
		Cultivated land density (C_8_)
	Economic development level (B_3_)	Per capita disposable income (C_9_)
		Spatial distribution difference index (C_10_)
		Per capita GDP (C_11_)
		Proportion of output value of tertiary industry (C_12_)
		Industrial added value (C_13_)
	Technology and management (B_4_)	Water consumption per unit of GDP (C_14_)
		Length of drainage pipe in built-up area (C_15_)
		Green coverage rate of built-up area (C_16_)
		Sewage treatment capacity (C_17_)
		Sewage treatment rate (C_18_)

**Table 2:** Importance scale values of judgment matrix.

Importance Scale Value	Meaning
1	Both elements are of equal importance
3	One element is slightly more important than the other
5	One element is more important than the other
7	One element is extremely important than the other
9	One element is absolutely important than the other
2,4,6,8	Represents the middle value of the above judgment

**Table 3:** Relative importance assessment criteria.

Linguistic Variable	Triangular Fuzzy Number
Equally important	(1,1,1)
Between equally important and slightly important	(1,2,3)
Slightly important	(1,3,5)
Between slightly important and important	(3,4,5)
Important	(3,5,7)
Between important and extremely important	(5,6,7)
Extremely important	(5,7,9)
Between extremely important and absolutely important	(7,8,9)
Absolutely important	(7,9,9)

**Table 4:** Judgment matrix of first expert on criterion layer.

Factors	Natural Conditions (B_1_)	Social Development Level (B_2_)	Economic Development Level (B_3_)	Technology and Management (B_4_)
Natural Conditions (B_1_)	1	1/5	1/3	1/7
Social Development Level (B_2_)	5	1	3	1/5
Economic Development Level (B_3_)	3	1/3	1	1/3
Technology and Management (B_4_)	7	5	3	1

**Table 5:** Information of experts.

Surname	Title	Work Unit	Expertise
Nie	Senior Engineer	Ministry of Water Resources, PRC	Technology Economics and Management in Water Resources
Zhang	Senior Engineer	Jiangsu Water Resources Department	Flood disaster prevention and control
Zhu	Senior Engineer	Jiangsu Water Resources Department	Flood disaster prevention and control
Tang	Vice Professor	Hohai University	Technology Economics and Management in Water Resources
Xu	Vice Professor	Hohai University	Technology Economics and Management in Water Resources
Shen	Lecturer	Hohai University	Environmental Science and Engineering
Liu	Lecturer	Nanjing Normal University	Technology Economics and Management in Water Resources

**Table 6:** Single weight values of indexes at all levels.

Judgment Matrix	Single Weight Values of the Index
K(A−B)	(0.219, 0.365, 0.182, 0.234)
$K\left(B_{1}-C\right)$	(0.244, 0.291, 0.181, 0.284)
$K\left(B_{2}-C\right)$	(0.299, 0.233, 0.166, 0.302)
$K\left(B_{3}-C\right)$	(0.213, 0.062, 0.372, 0.158, 0.194)
$K\left(B_{4}-C\right)$	(0.251, 0.277, 0.124, 0.205, 0.142)
K(A−C)	(0.053, 0.064, 0.040, 0.062, 0.109, 0.085, 0.060, 0.110, 0.039, 0.011, 0.068, 0.029, 0.035, 0.059, 0.065, 0.029, 0.048, 0.033)

**Table 7:** Results of initial allocations of flood drainage rights in each administrative region.

Index	C_1_	C_2_	C_3_	C_4_	C_5_	C_6_	C_7_	C_8_	C_9_	C_10_	C_11_	C_12_	C_13_	C_14_	C_15_	C_16_	C_17_	C_18_	Total Weight
Weight	0.053	0.064	0.040	0.062	0.109	0.085	0.060	0.110	0.039	0.011	0.068	0.029	0.035	0.059	0.065	0.029	0.048	0.033	1.000
Wuxi	0.118	0.124	0.085	0.068	0.134	0.130	0.076	0.105	0.128	0.125	0.156	0.121	0.141	0.129	0.184	0.131	0.085	0.129	0.121
Changzhou	0.118	0.124	0.080	0.093	0.102	0.123	0.014	0.146	0.116	0.125	0.137	0.120	0.087	0.122	0.083	0.132	0.065	0.128	0.107
Suzhou	0.096	0.124	0.159	0.057	0.117	0.130	0.171	0.078	0.140	0.124	0.158	0.121	0.235	0.128	0.146	0.126	0.161	0.125	0.127
Zhenjiang	0.129	0.124	0.070	0.140	0.079	0.120	0.036	0.172	0.103	0.124	0.122	0.111	0.056	0.133	0.028	0.131	0.031	0.125	0.104
Hangzhou	0.152	0.124	0.305	0.285	0.054	0.131	0.263	0.054	0.138	0.126	0.131	0.148	0.123	0.129	0.120	0.112	0.112	0.125	0.137
Jiaxing	0.139	0.135	0.078	0.108	0.104	0.110	0.210	0.207	0.111	0.127	0.092	0.104	0.065	0.120	0.061	0.117	0.036	0.118	0.119
Huzhou	0.129	0.124	0.107	0.224	0.049	0.106	0.131	0.111	0.103	0.127	0.081	0.112	0.033	0.110	0.031	0.130	0.017	0.127	0.100
Shanghai	0.120	0.122	0.116	0.026	0.361	0.150	0.099	0.128	0.163	0.122	0.123	0.163	0.260	0.130	0.346	0.119	0.492	0.124	0.184

**Table 8:** Environmental Gini coefficients based on land area.

Administrative Region	Land Area (10,000 km^2^)	Allocation Weight of Flood Drainage Rights	Flood Drainage Rights Per Unit Land Area	Percentage (%)		Cumulative Percentage (%)	
				Land Area	Flood Drainage Rights	Land Area	Flood Drainage Rights
Hangzhou	1.660	0.137	0.083	30.463	13.712	30.463	13.712
Suzhou	0.866	0.127	0.147	15.891	12.734	46.354	26.447
Huzhou	0.582	0.100	0.172	10.683	10.001	57.037	36.448
Changzhou	0.437	0.107	0.245	8.029	10.719	65.066	47.167
Wuxi	0.463	0.121	0.261	8.494	12.089	73.560	59.257
Zhenjiang	0.384	0.104	0.271	7.049	10.416	80.609	69.672
Jiaxing	0.422	0.119	0.283	7.752	11.935	88.361	81.607
Shanghai	0.634	0.184	0.290	11.639	18.393	100.000	100.000

**Table 9:** Allocation results of flood drainage rights.

Region	Initial Allocation Weight	Final Allocation Weight	Adjustment Amount	Adjusted Proportion (%)
Wuxi	0.121	0.107	−0.014	−11.657
Changzhou	0.107	0.082	−0.025	−23.603
Suzhou	0.127	0.144	0.017	13.461
Zhenjiang	0.104	0.089	−0.016	−14.910
Hangzhou	0.137	0.128	−0.009	−6.620
Jiaxing	0.119	0.097	−0.022	−18.332
Huzhou	0.100	0.097	−0.003	−2.878
Shanghai	0.184	0.256	0.072	38.936

**Table 10:** Optimization results of environmental Gini coefficients.

Environmental Gini Coefficient	$G_{L}$	$G_{P}$	$G_{G}$	$G_{S}$	∑G
Initial environmental Gini coefficient	0.250	0.286	0.315	0.439	1.290
Optimized environment Gini coefficient	0.281	0.183	0.206	0.336	1.006
